# High spatial resolution gene expression profiling and characterization of neuroblasts migrating in the peri-injured cortex using photo-isolation chemistry

**DOI:** 10.3389/fnins.2024.1504047

**Published:** 2025-01-07

**Authors:** Takuya Miyamoto, Kazuya Kuboyama, Mizuki Honda, Yasuyuki Ohkawa, Shinya Oki, Kazunobu Sawamoto

**Affiliations:** ^1^Department of Developmental and Regenerative Neurobiology, Institute of Brain Science, Nagoya City University Graduate School of Medical Sciences, Nagoya, Japan; ^2^Department of Drug Discovery Medicine, Kyoto University Graduate School of Medicine, Kyoto, Japan; ^3^Laboratory of Molecular and Cellular Physiology, Graduate School of Integrated Sciences for Life, Hiroshima University, Hiroshima, Japan; ^4^Division of Transcriptomics, Medical Institute of Bioregulation, Kyushu University, Fukuoka, Japan; ^5^Institute of Resource Development and Analysis, Kumamoto University, Kumamoto, Japan; ^6^Division of Neural Development and Regeneration, National Institute for Physiological Sciences, Okazaki, Japan

**Keywords:** postnatal neurogenesis, ventricular-subventricular zone, neuroblasts, spatial transcriptome, neuronal regeneration

## Abstract

In the ventricular-subventricular-zone (V-SVZ) of the postnatal mammalian brain, immature neurons (neuroblasts) are generated from neural stem cells throughout their lifetime. These V-SVZ-derived neuroblasts normally migrate to the olfactory bulb through the rostral migratory stream, differentiate into interneurons, and are integrated into the preexisting olfactory circuit. When the brain is injured, some neuroblasts initiate migration toward the lesion and attempt to repair the damaged neuronal circuitry, but their low regeneration efficiency prevents functional recovery. Elucidation of the molecular basis of neuroblast migration toward lesions is expected to lead to the development of new therapeutic strategies for brain regenerative medicine. Here, we show gene expression profiles of neuroblasts migrating in the peri-injured cortex compared with those migrating in the V-SVZ using photo-isolation chemistry, a method for spatial transcriptome analysis. Differentially expressed gene analysis showed that the expression levels of 215 genes (97 upregulated and 118 downregulated genes) were significantly different in neuroblasts migrating in the peri-injured cortex from those migrating in the V-SVZ. Gene Ontology analysis revealed that in neuroblasts migrating in the peri-injured cortex, expression of genes involved in regulating migration direction and preventing cell death was upregulated, while the expression of genes involved in cell proliferation and maintenance of the immature state was downregulated. Indeed, neuroblasts migrating in the peri-injured cortex had significantly lower Cyclin D2 mRNA and Ki67 protein expression levels than those in the V-SVZ. In the injured brain, amoeboid microglia/macrophages expressed transforming growth factor-*β* (TGF-*β*), and neuroblasts migrating in the peri-injured cortex expressed TGF-*β* receptors. Experiments using primary cultured neuroblasts showed that application of TGF-*β* significantly decreased proliferating cells labeled with BrdU. These data suggest that the proliferative activity of neuroblasts migrating toward lesions is suppressed by TGF-*β* secreted from cells surrounding the lesion. This is the first comprehensive study characterizing the gene expression profiles of neuroblasts migrating in the peri-injured cortex.

## 1 Introduction

In the postnatal brains of mammals, including humans, the ventricular-subventricular-zone (V-SVZ) and hippocampal dentate gyrus are neurogenesis niches where neural stem cells (NSCs) in an active state are localized (Gage, 2000; Alvarez-Buylla and Garcia-Verdugo, 2002). Activated NSCs generate migratory immature neurons (neuroblasts) through transient amplifying progenitor cells. In the V-SVZ, newly generated neuroblasts are integrated within homogeneous chain-like clusters and continuously migrate to the olfactory bulb (OB) through the rostral migratory stream (RMS). Upon reaching the OB, most neuroblasts differentiate into mature granule cells and connect with preexisting OB circuits as inhibitory interneurons (Lois and Alvarez-Buylla, 1994; Bressan and Saghatelyan, 2021; Nakajima et al., 2021). Uniquely, neuroblasts actively proliferate, even during the migration process from the V-SVZ to the OB, while remaining in an immature state (Menezes et al., 1995; Ikeda et al., 2010).

After brain injury such as trauma or stroke, the neurogenic activity of NSCs in the V-SVZ is enhanced, thereby promoting the generation of neuroblasts (Arvidsson et al., 2002; Zhang et al., 2002; Yamashita et al., 2006; Alagappan et al., 2009; Saha et al., 2013; Goodus et al., 2015). Some of these neuroblasts respond to soluble factors secreted from the injured area and initiate migration toward the lesion (Mundim et al., 2019; Nakajima et al., 2021). After reaching the peri-injured area, migratory neuroblasts mature and replace the lost neurons (Kaneko et al., 2018). Studies have indicated that neuroblasts generated in the V-SVZ have potential as a source of endogenous new neurons for brain regenerative medicine. In practice, however, only a few neuroblasts reach the injured area and are integrated into preexisting circuits, thus limiting their contribution to the recovery of brain function. Numerous recent studies have shown that interventions that promote neuroblast migration toward peri-injured areas support recovery of brain function (Kaneko et al., 2018; Jinnou et al., 2018; Ohno et al., 2023; Nakajima et al., 2024; Matsumoto et al., 2024). However, because the biology of neuroblasts migrating toward lesions is not fully understood, there is insufficient information for these intervention strategies.

The single-cell RNA sequencing technique is a powerful tool that allows comprehensive molecular profiling of specific cell types. Although single-cell RNA sequencing has the experimental limitation of not retaining the spatial information of individual cells, spatial transcriptome techniques overcome this problem. Spatial transcriptome techniques include the direct capture of mRNA from tissue sections using glass slides with spatially barcoded oligonucleotides (e.g., 10x Genomics Visium), as well as laser microdissection and photo-isolation chemistry (PIC) techniques, which increase spatial resolution (Cheng et al., 2023). Using PIC, it is possible to identify cell types based on cell markers and morphological features using immunohistochemical staining and reporter transgenic mice and then prepare a library of genes expressed in those identified cells (Honda et al., 2022).

Transforming growth factor-*β* (TGF-*β*) has been reported as a transforming factor of normal rat fibroblasts (Roberts et al., 1981). Studies have revealed that TGF-*β* inhibits proliferation and promotes fibrosis and the epithelial-to-mesenchymal transition (Morikawa et al., 2016). TGF-*β* has also been reported to be secreted by activated microglia and/or invading macrophages in the pathological process of stroke in humans and rats (Lindholm et al., 1992; Krupinski et al., 1996; Lehrmann et al., 1998). Additionally, TGF-*β* has been reported to inhibit neural stem/progenitor cell proliferation during development and after birth (Wachs et al., 2006; Vogel et al., 2010). However, the effects of TGF-*β* on postnatal migrating neuroblasts have not been investigated.

In this study, we performed PIC in a mouse model of cryogenic cortical injury and revealed differences in gene expression profiles between neuroblasts migrating in the V-SVZ and those migrating in the peri-injured cortex. Our results suggest that neuroblasts migrating in the peri-injured cortex exhibit less proliferative activity, possibly because of TGF-*β* signaling.

## 2 Methods

### 2.1 Animals

ICR mice were purchased from Japan SLC. *Dcx*-EGFP mice (Gong et al., 2003) were provided by the Mutant Mouse Research Resource Center (MMRRC. RRID: MMRRC_000244-MU). All animals were maintained at a temperature of 22 ± 1°C with a 12-h light/dark cycle (lights on between 08:00 and 20:00) and had *ad libitum* access to food and water. All animal experiments were performed in accordance with the guidelines and regulations of Nagoya City University (Approval No. 21–028).

### 2.2 Reagents and antibodies

Recombinant human TGF-*β*1 (rhTGF-*β*1) was purchased from Abcam (cat# ab50036), reconstituted in 10 mM citric acid (pH 3.0) to a concentration of 5 μg/100 μL, and stored at −80°C. The following commercially available primary antibodies were used in the present study: rat anti-green fluorescent protein (GFP; 1:500, Nacalai Tesque, cat# 04404–84); rabbit anti-GFP (1:1000, MBL, cat# 598); guinea pig anti-Dcx (1:200, Millipore, cat# AB2253); rat anti-Ki67 (1:500, eBioscience, cat# 14–5698-82); goat anti-Iba1 (1:500, Abcam, cat# ab5076); rabbit anti-TGF-*β*1 (1:500, Abcam, cat# ab215715); rat anti-TGF-*β*RI (1:100, Santa Cruz Biotechnology, cat# sc-101574), mouse anti-TGF-*β*RII (1:100, Santa Cruz Biotechnology, cat# sc-17791); rat anti-BrdU (1:500, Abcam, cat# ab6326); and rabbit anti-Pax6 (1:100, Covance, cat# 901301). Nuclei were stained with Hoechst 33342 (1:10000, Sigma-Aldrich).

### 2.3 Neonatal cerebral cortical cryogenic injury model

Postnatal day 2 mice were subjected to cerebral cortical cryogenic injury as described previously (Jinnou et al., 2018; Nakajima et al., 2024). Briefly, the mice were placed in a stereotaxic instrument (David Kopf Instruments) and anesthetized with 1% isoflurane in oxygen (0.3 L/min). Then, the skull was exposed through a scalp incision. A pre-chilled hexagonal 1.2 mm-wide wrench was used as a probe and prepared using liquid nitrogen. The metal probe was placed on the exposed right skull (0.5 mm anterior and 1.2 mm right lateral to the true lambda) for 10 s. The procedure was repeated three times with an interval of 5 s to re-chill the probe. The scalp was sutured, and the mice were placed in a heated recovery box for 15 min and then returned to their home cage. At 7 days post-injury, mice were fixed by transcardiac perfusion with 4% paraformaldehyde (PFA) in 0.1 M phosphate buffer and postfixed 4 h or overnight in the same fixative at 4°C.

### 2.4 Primary V-SVZ-derived neuroblast culture

*In vitro* culture of V-SVZ-derived neuroblasts was performed as described previously (Sawada et al., 2020). Briefly, the V-SVZ tissues were dissected from postnatal day 0–1 pups and dissociated using trypsin–EDTA (Invitrogen). The cells were washed twice in L-15 medium (Invitrogen) containing 40 μg/mL DNaseI (Roche Diagnostics) and seeded on Matrigel (BD Biosciences)-coated cover glasses (Matsunami) with Neurobasal medium (Invitrogen) containing 2% B-27 Supplement (Invitrogen), 2 mM GlutaMAX (Invitrogen), 50 U/mL penicillin–streptomycin (Invitrogen), and 5 μM BrdU (Sigma-Aldrich). rhTGF-β1 was added to the culture medium at a final concentration of 0, 0.5, or 5 ng/mL. At 3 days *in vitro*, cells were fixed with 4% PFA in 0.1 M phosphate buffer for 30 min at room temperature (RT) and subjected to immunocytochemistry.

### 2.5 Spatial transcriptomic analysis (PIC)

After fixation in 4% PFA and postfixation for 4 h, brain samples were incubated in 15 and 30% sucrose solutions, encapsulated in OCT compound (Sakura Finetek Japan), and then cooled using dry ice and isopentane. Serial brain slices (around 0.5 mm anterior to the bregma, slices span: 280 μm) were obtained at a thickness of 10 μm using a cryostat (CryoStar NX70, Epredia). The PIC technique was then performed as described previously (Honda et al., 2022) Briefly, reverse transcription was performed by adding ultraviolet (UV)-responsive 6-nitropiperonyloxymethyl-caged reverse transcription primers containing the T7 promotor, unique molecular identifiers (UMIs), multiple barcodes, and a polyT sequence onto the sections. Immunofluorescence was then performed to visualize regions of interest for subsequent UV irradiation. To cleave 6-nitropiperonyloxymethyl moieties from reverse transcription primers, the regions of interest were irradiated with UV light for 3 min with a Digital Micromirror Device (Polygon 1,000-G; Mightex Systems). The total tissue lysate was then collected and purified using 20 mg/mL proteinase K. Second-strand DNA was synthesized using the nick translation method. For *in vitro* transcription reactions, synthesized cDNAs were transcribed into RNAs using a T7 transcription kit. The amplified RNAs were further reverse transcribed, followed by paired-end sequencing on an Illumina platform. The sequences were separated by the sample barcodes using UMI-tools and mapped to the reference genome using HISAT2. UMI-tools and feature Counts were used to generate the UMI count data assigned to genes. Differentially expressed genes (DEGs) were extracted using DESeq2 [false discovery rate (FDR) = 0.1]. DESeq2 was also used to transform the count data into regularized log data before performing principal component analysis using the R prcomp function. Gene Ontology (GO) analyses were performed using Metascape (Zhou et al., 2019).^1^

### 2.6 Immunohistochemistry and immunocytochemistry

The fixed brain was sectioned coronally into 60-μm serial slices (around 0.5 mm anterior to the bregma, slices span: 300 μm) using a vibratome (VT-1200S, Leica) or into 10-μm slices using a cryostat and incubated for 30 min at RT in blocking solution (10% normal donkey serum and 0.2% TritonX-100 in PBS). The brain sections or fixed cells were then incubated overnight at 4°C with the primary antibodies. Bound primary antibodies on samples were visualized with Alexa Fluor-conjugated secondary antibodies (1:1000, Invitrogen) or biotin-conjugated antibodies (1:1000, Jackson ImmunoResearch) along with the Vectastain Elite ABC kit (Vector Technology) and Tyramide Signal Amplification (Thermo Fisher Scientific) according to the standard procedure.

### 2.7 Fluorescence *in situ* hybridization (RNAscope)

Fluorescence *in situ* hybridization was performed using the RNAscope multiplex assay (Advanced Cell Diagnostics) according to the manufacturer’s protocol (Wang et al., 2012). Briefly, after OCT compound removal, primary antibodies were applied to 10-μm thick cryostat serial sections (around 0.5 mm anterior to the bregma, slices span: 280 μm) for immunohistochemistry. Then, the sections were fixed using 4% PFA at RT for 30 min, dehydrated with ethanol, treated with 1% hydrogen peroxide at RT for 30 min, and boiled in RNAscope Target Retrieval Reagent (Advanced Cell Diagnostics) at 98°C for 15 min. Next, the sections were hybridized with the probes for 2 h at 40°C. The following commercially available primary antibodies were used in the present study: Ccnd2 probe (Mm-Ccnd2, Advanced Cell Diagnostics, cat# 433211); Sez6l probe (Mm-Sez6l, Advanced Cell Diagnostics, cat# 492631); and Dner probe (Mm-Dner, Advanced Cell Diagnostics, cat# 413951). The sections were then washed and hybridized with amplifiers and visualized using Opal620 (1:1000, Akoya Bioscience). Immediately after this process, secondary antibodies were applied as described in the immunohistochemistry and immunocytochemistry section.

### 2.8 Confocal image acquisition and quantification

Images were acquired using an FV3000 confocal laser-scanning microscope (Evident) with a 20× objective lens (NA 0.8) or 40× objective lens (NA 1.4). To count the *Ccnd2-*, *Sez6l*-, and *Dner*- mRNA puncta in nuclei, Dcx-positive cells were randomly selected from the dorsal corner of the V-SVZ and migratory zone. All Dcx-positive cells in the field of view were selected to count BrdU-incorporating cells among Dcx-positive cells *in vitro*.

### 2.9 Statistical analysis

All statistical tests were two-tailed and analyzed using EZR (Kanda, 2013). The data distribution was analyzed using the Kolmogorov–Smirnov test. Comparisons between two groups were performed using an unpaired t-test. Comparisons among multiple groups were performed using the Kruskal–Wallis test followed by a post-hoc Bonferroni test or one-way ANOVA followed by a post-hoc Bonferroni test. All numerical data are presented as the mean and SEM, and *p*-values less than 0.05 were considered statistically significant.

## 3 Results

### 3.1 Selection of neuroblasts to be UV-irradiated for PIC analysis

**Figure 1:**
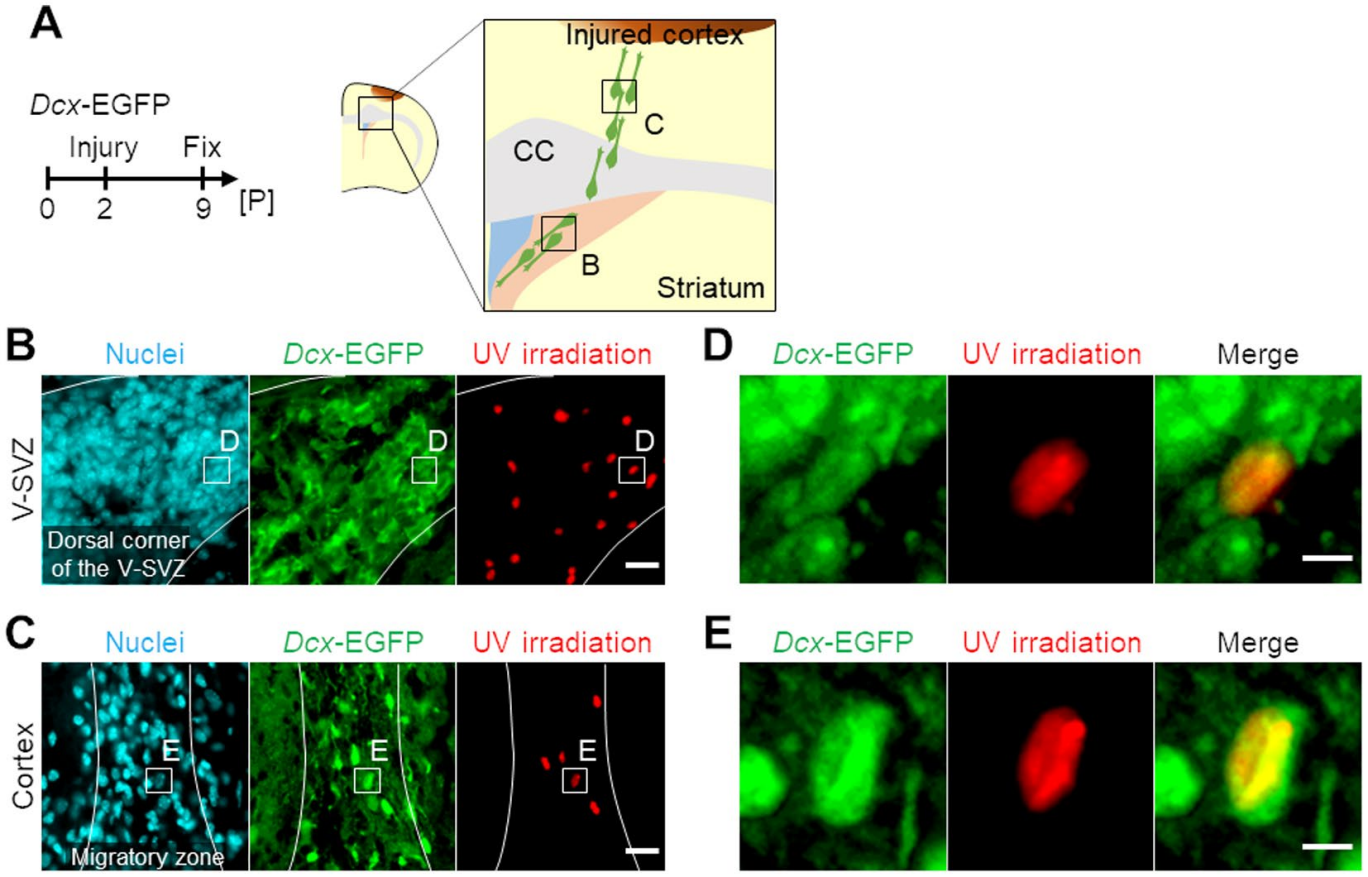
Photo-isolation chemistry (PIC) analyses of neuroblasts migrating in the ventricular-subventricular-zone (V-SVZ) and the peri-injured cortex in neonatal cerebral cortex cryogenic injury model mice. **(A)** Experimental schedule and schematic image of a coronal section of the cryogenic injured mouse brain. *Dcx*-EGFP mice were subjected to cryogenic brain injury at postnatal day 2 (P2) and sacrificed at P9, 7 days post-injury. CC, corpus callosum. **(B,C)** Spatial-specific ultraviolet (UV) irradiation for PIC analyses. Images of immunohistochemical staining of *Dcx*-EGFP (green) with nuclei (cyan) and areas of UV irradiation (red) in the V-SVZ **(B)** and the peri-injured cortex **(C)** of the injured brain are shown. **(D,E)** Magnified images of the V-SVZ **(D)** and the peri-injured cortex **(E)** in panel **B** and **C**, respectively. Scale bars, 20 μm **(B,C)**, 5 μm **(D,E)**.

To compare gene expression profiles of neuroblasts migrating through the V-SVZ (hereafter referred to as “V-SVZ neuroblasts”) and those migrating in the peri-injured cortex (hereafter referred to as “cortical neuroblasts”), the cerebral cortex of *Dcx*-EGFP mice, in which neuroblasts were labeled with EGFP, was cryoinjured, and brain sections were prepared for spatial transcriptome analysis (see Materials and Methods; Figure 1A). We identified V-SVZ neuroblasts or cortical neuroblasts as GFP-positive cells localized in the V-SVZ (Figure 1B) or peri-injured cortex (Figure 1C), respectively, and irradiated their Hoechst-labeled nuclei with UV light. GFP-positive nuclei were randomly selected when irradiating V-SVZ neuroblasts (Figure 1D), but among cortical neuroblasts, GFP-positive nuclei that did not overlap with surrounding nuclei and were oval in the direction of migration were selected (Figure 1E; Supplementary Figure 1).

### 3.2 DEGs and GO analyses of genes expressed in cortical and V-SVZ neuroblasts

**Figure 2:**
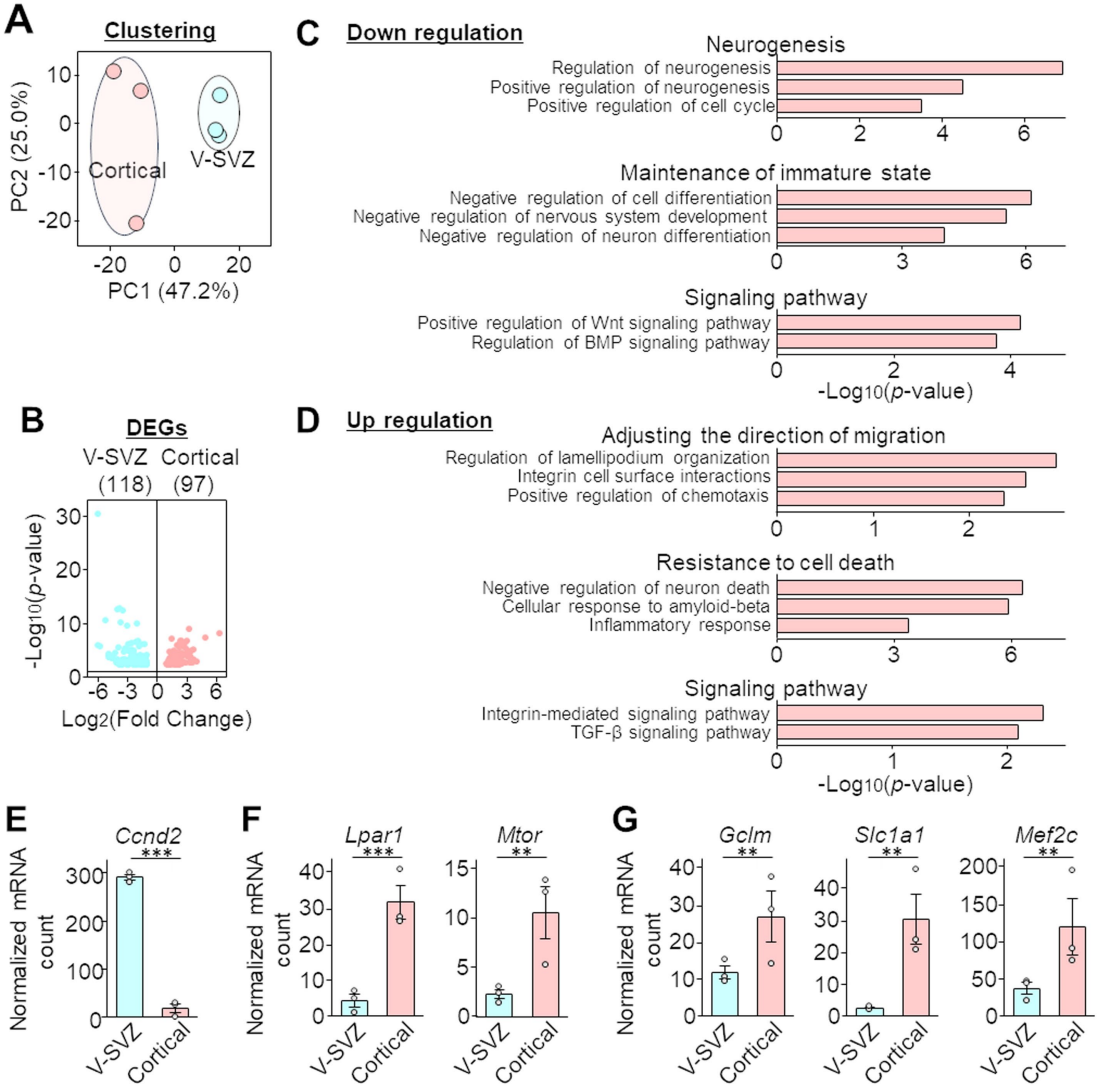
Differentially expressed genes (DEGs) and Gene Ontology (GO) analyses of the PIC RNA sequencing dataset of neuroblasts migrating in the V-SVZ and peri-injured cortex. **(A)** Two-dimensional principal component analyses (PCA) of the gene expression profiles of PIC analyses data. Blue or red circles indicate three mice. The horizontal and vertical axes of the graph indicate the percentage of the first principal component (PC1) and the second principal component (PC2), respectively. Sequencing quality data are shown in Supplementary Figure 2. **(B)** Volcano plot of DEG analysis. The horizontal and vertical axes indicate the expression ratio and *p*-value, respectively. All information of significant genes with *p*-values less than 0.05 in the DEG analysis is shown in the Supplementary Table 1, and the validation of PIC RNA sequencing is shown in Supplementary Figure 3. **(C,D)** Selected GO terms with downregulated **(C)** and upregulated **(D)** expression in cortical neuroblasts compared with that in V-SVZ neuroblasts. The horizontal axes indicate the *p*-value. **(E–G)** Normalized mRNA counts for Ccnd2 (associated with the GO term neurogenesis, **E**), Lpar1 and Mtor (migratory orientation, **F**), and Gclm, Slc1a1, and Mef2c (inflammation and resistance, **G**). Gray circles indicate individual samples. Graphs represent the mean and SEM of three mice. **p < 0.01, ***p < 0.001, Student’s *t*-test.

V-SVZ and cortical neuroblasts were collected from four brain slices per mouse, for a total three mice each (50, 50, and 50 nuclei from the V-SVZ; 44, 45, and 50 nuclei from the peri-injured cortex). There was no significant bias in the number of reads and assigned reads/UMIs (Supplementary Figures 2A,B). In the V-SVZ neuroblast-derived samples, 12,959, 11,350, and 14,314 genes (average 12,874 genes) were detected, with UMI counts per cell of 2,788, 1,966, and 4,051 (average 2,935), respectively (Supplementary Figures 2C,D). In contrast, in the cortical neuroblast-derived samples, 10,419, 3,544, and 8,313 genes (average 7,425 genes) were detected, with UMI counts per cell of 1,489, 205, and 728 (average 807), respectively (Supplementary Figures 2C,D). The principal component analysis results of the gene expression profiles showed that the clusters of cortical neuroblast-derived and V-SVZ neuroblast-derived samples were clearly separable according to their origin (Figure 2A). DEG analysis showed that a total of 215 genes were significantly differentially expressed (FDR = 0.1), with 97 genes upregulated in cortical neuroblasts and 118 genes upregulated in V-SVZ neuroblasts (Figure 2B). To confirm the veracity of the DEG data obtained, immunostaining and FISH were performed on the three molecules that showed moderate changes in expression. Consistent with the DEG dataset, immunoreactivity of the Pax6 protein (Kohwi et al., 2005) was significantly downregulated in cortical neuroblasts compared to V-SVZ neuroblasts (Supplementary Figures 3A–C). In contrast, the numbers of fluorescent puncta for *Sez6l-* and *Dner*-mRNA were significantly increased in cortical neuroblasts (Supplementary Figures 3D–I). These results indicate clear differences in the gene expression profiles between cortical and V-SVZ neuroblasts.

GO analysis was then performed on the 97 DEGs that had significantly higher expression and the 118 DEGs that had significantly lower expression in cortical neuroblasts than in V-SVZ neuroblasts. Genes that were downregulated in cortical neuroblasts showed terms related to neurogenesis, maintenance of the immature state, and the Wnt and BMP signaling pathways (Figure 2C). However, genes that were upregulated in cortical neuroblasts had terms related to adjusting the direction of migration, resistance to cell death, and the integrin and TGF-*β* signaling pathways (Figure 2D). For example, Ccnd2 (which was associated with the GO term neurogenesis) was significantly downregulated in cortical neuroblasts (Figure 2E). However, Lpar1 and Mtor (Figure 2F, associated with the GO term migratory orientation) and Gclm, Scl1a1, and Mef2c (Figure 2G, associated with the GO terms inflammation and resistance) were significantly upregulated in cortical neuroblasts. These results suggest that cortical and V-SVZ neuroblasts have distinct gene expression properties, and thus these cells exhibit different characteristics.

### 3.3 Proliferative activity of migratory neuroblasts in the peri-injured cortex

**Figure 3:**
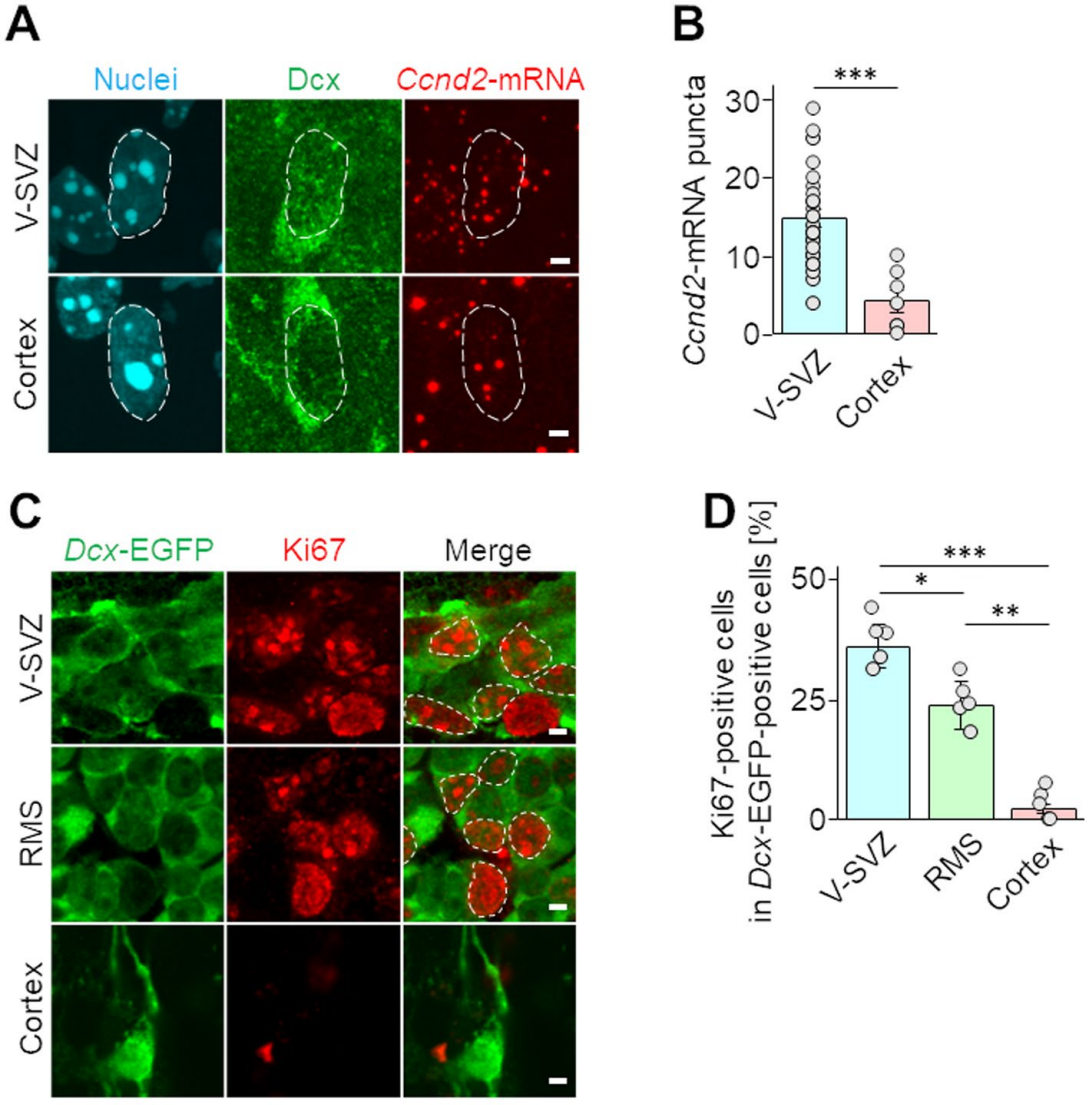
Proliferative activities of neuroblasts migrating in the V-SVZ and peri-injured cortex. **(A,B)** Wild-type mice were cryoinjured at postnatal day 2 and fixed 7 days later at postnatal day 9. Fluorescence images show *in situ* hybridization signals of *Ccnd2*-mRNA (red) and immunohistochemical staining for Dcx (green) along with nuclei (cyan, dashed line) in V-SVZ and cortical neuroblasts **(A)**. The numbers of *Ccnd2*-mRNA puncta distributed in the nuclei in Dcx positive cells were counted **(B)**. Each graphs represents the mean and SEM of 42 cells in the V-SVZ and 15 cells in the cortex of 5 mice (2–3 slices per mouse). ***p < 0.001, Student’s t-test. **(C,D)** Images of immunohistochemical staining of Ki67 (red, dashed line) and EGFP (green) in neuroblasts migrating through the V-SVZ, rostral migratory stream (RMS), and peri-injured cortex in *Dcx*-EGFP mice at 7 days post cryogenic injury **(C)**. The percentages of cells expressing Ki67 among *Dcx*-EGFP cells were calculated **(D)**. The graph represents the mean and SEM of 5 mice (5 slices per mouse). *p < 0.05, **p < 0.01, ***p < 0.001, Kruskal–Wallis with Bonferroni test. Scale bars, 2 μm **(A,C)**.

To verify the downregulation of Ccnd2 in cortical neuroblasts detected by PIC, *Ccnd2*-mRNA expression was visualized by fluorescence *in situ* hybridization. In brains at 7 days after injury, *Ccnd2*-mRNA signaling puncta in the nuclei of Dcx-positive neuroblasts were significantly reduced in cortical neuroblasts compared with those in V-SVZ neuroblasts (Figures 3A,B). The proliferative activity of neuroblasts was also assessed by immunohistochemical staining for Ki67, which is expressed during all active phases of cell cycle progression (G1, S, G2, and M), but not in the G0 resting phase (Zacchetti et al., 2003). Approximately 37% of V-SVZ neuroblasts and 24% of RMS neuroblasts were Ki67-positive, whereas only approximately 2% of cortical neuroblasts were Ki67-positive (Figures 3C,D). These results suggest that cortical neuroblasts have suppressed proliferative activity.

### 3.4 TGF-*β* expression in the peri-injured cortex of neonatal mice with cryogenic brain injury

**Figure 4:**
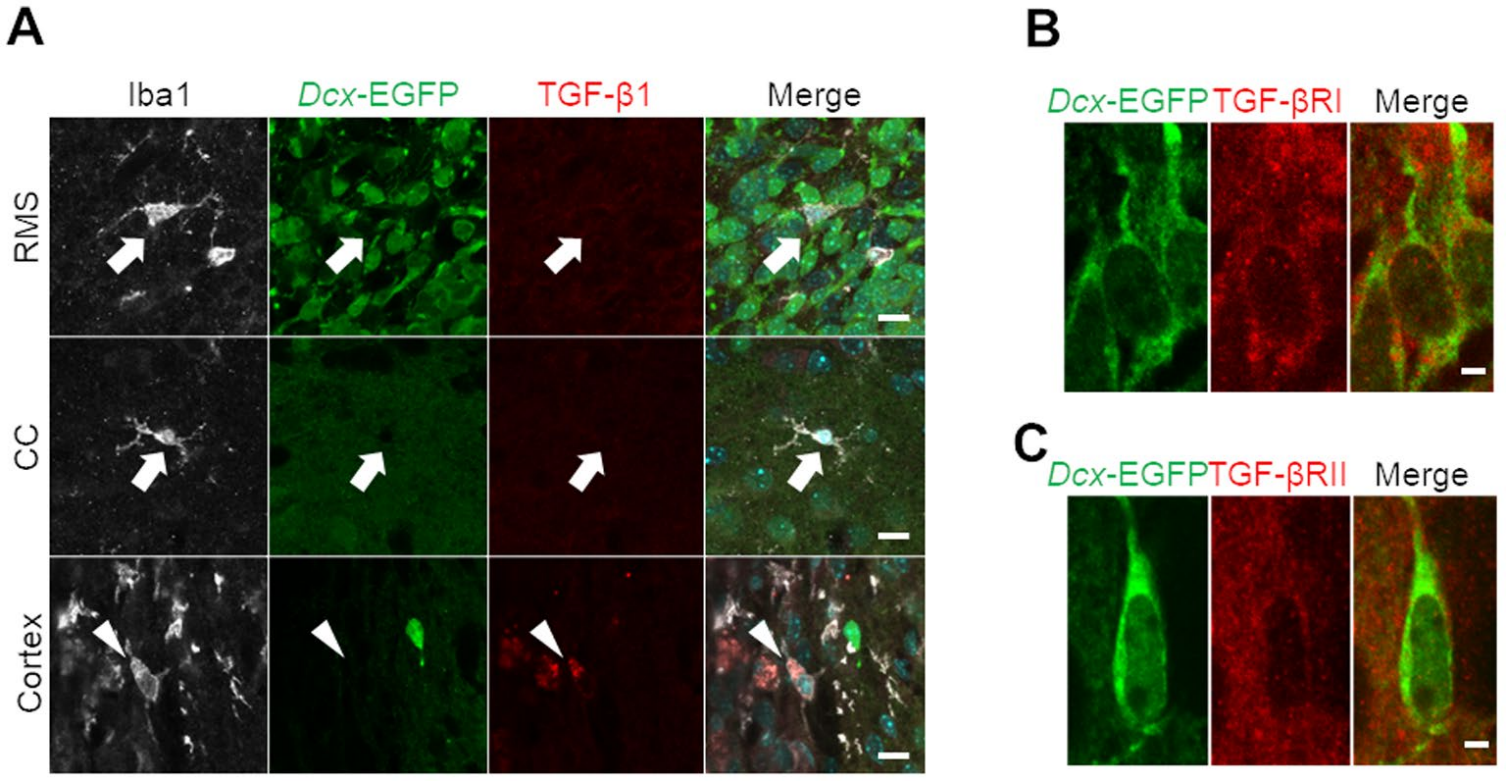
Transforming growth factor (TGF)-*β* secretion from microglia/macrophages and TGF-*β* receptor expression in neuroblasts in the cryoinjured brain. **(A)** *Dcx*-EGFP mice were cryoinjured at postnatal day 2 and fixed 7 days later at postnatal day 9. Images show immunohistochemical staining for Iba1 (white), *Dcx*-EGFP (green), and TGF-*β*1 (red) in the RMS, corpus callosum (CC), and peri-injured cortex. Arrows indicate TGF-*β*1-negative, Iba1-positive cells, and arrowheads indicate TGF-*β*1-positive, Iba1-positive cells. **(B,C)** Images of immunohistochemical staining for *Dcx*-EGFP (green) and TGF-*β* receptors (type I in panel **B** and type II in panel **C**, red) in the peri-injured cortex. Scale bars, 10 μm **(A)**, 2 μm **(B)**.

Because TGF-*β* is a representative inhibitory factor of proliferation that has been extensively studied (Morikawa et al., 2016), we focused on TGF-*β* signaling pathway-related genes, among which expression was upregulated in cortical neuroblasts in our GO analysis (Figure 2D). In a neonatal mouse model of cortex injury, Iba1-positive microglia/macrophages localized in areas away from the injured area, such as the RMS and corpus callosum, exhibited non-activated ramified morphology (Vidal-Itriago et al., 2022), and did not express TGF-*β*1 (Figure 4A). In contrast, in the peri-injured cortex, Iba1-positive cells had activated amoeboid morphology and expressed TGF-*β*1 protein (Figure 4A). To determine whether neuroblasts could respond to secreted TGF-*β*1, cortical neuroblasts were stained for TGF-*β* receptors. *Dcx*-EGFP-positive neuroblasts expressed both type I and type II TGF-*β* receptors (Figures 4B,C). These results suggest that TGF-*β* is present in the peri-injured cortex and that it can affect neuroblasts.

### 3.5 Effect of TGF-*β* on proliferative activity of neuroblasts

**Figure 5:**
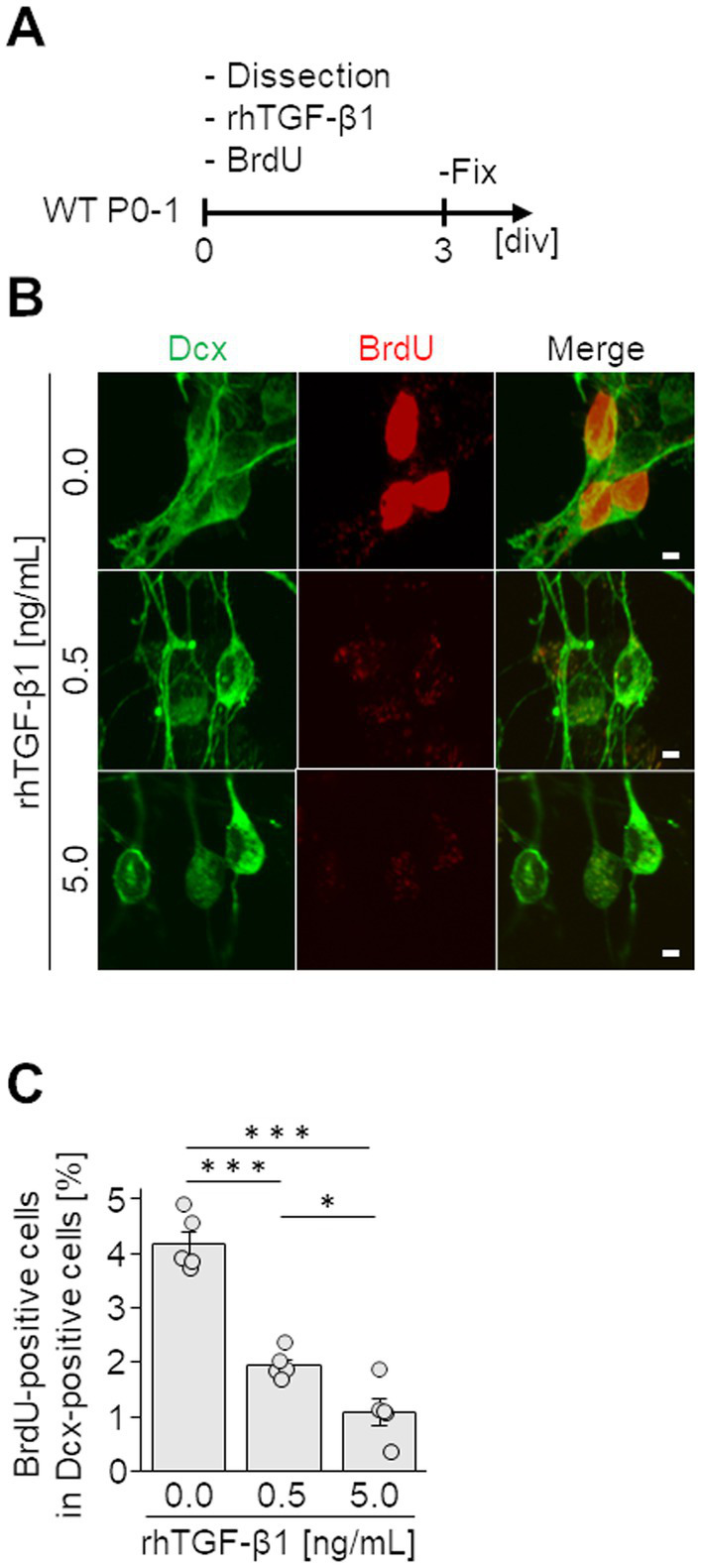
Effect of TGF-*β* on neuroblasts. **(A)** Experimental schedule. Neuroblasts derived from the V-SVZ (12 pups per 24-well plate) were cultured for 3 days (3 div) with various concentrations of recombinant human TGF-*β*1 (rhTGF-*β*1) and BrdU. **(B)** Images of immunocytochemical staining for Dcx (green) and BrdU (red). **(C)** Percentages of BrdU-incorporating Dcx-positive neuroblasts were counted. Data represent the mean and SEM of 5 independent cultures analyzing 50–100 cells each. *p < 0.05, ***p < 0.001, one-way ANOVA with Bonferroni test. Scale bars, 2 μm.

Finally, to investigate the effect of TGF-*β* on the proliferative activity of neuroblasts, V-SVZ-derived neuroblasts were cultured with various concentrations (0, 0.5, and 5.0 ng/mL) of rhTGF-*β*1. The proliferative activity of neuroblasts was assessed by BrdU incorporation into the nucleus. In the control group, more than 4% of Dcx-positive neuroblasts were proliferating cells labeled with BrdU. In contrast, in the groups supplemented with 0.5 and 5.0 ng/mL of rhTGF-*β*1, only less than 2% of Dcx-positive neuroblasts were positive for BrdU, indicating that proliferating cells were significantly reduced compared with those in the control group (Figures 5B,C). These results suggest that the proliferative activity of neuroblasts is suppressed by TGF-*β*1.

## 4 Discussion

In the present study, a spatial transcriptomic analysis method, PIC, was used to compare migratory neuroblasts in the V-SVZ and peri-injured cortex and to identify differences in their gene expression profiles. In cortical neuroblasts, reduced cell proliferation involving TGF-*β* secreted from activated microglia/macrophages was revealed by GO analysis of DEGs and immunohistochemical studies. These results will provide insights into the characterization of neuroblasts in postnatally injured brains.

### 4.1 PIC is useful for spatial transcriptomics analysis of injured/diseased tissues

Because lesions and their surrounding areas in the injured/diseased brain, including a variety of injured or dead cells and recruited immune cells, show more complex structures than those in intact areas (Meirelles et al., 2017; Yu et al., 2022), high spatial resolutions are required for analyses targeting these lesions. Previously, several spatial transcriptomics analyses of the injured/diseased brain have been performed using spatial capture technology (e.g., 10x Visium) or laser microdissection (Boone et al., 2013; Li et al., 2023; Zheng et al., 2023). However, these techniques had several disadvantages, such as the large size of the analysis unit, which contains multiple cells, and burned regions that cannot be analyzed. In contrast, the PIC method we used can capture samples with nanometer-level spatial resolution and without loss of tissue (Honda et al., 2022). Moreover, PIC can detect gene expression with higher sensitivity than single-cell RNA sequencing or other spatial transcriptome technologies (Supplementary Figure 2; Honda et al., 2022). Using PIC, we succeeded in obtaining small differences in the gene expression profiles of V-SVZ and cortical neuroblasts in the injured brain by precisely segmenting the nuclei of cells of interest based on the labeled proteins and cell morphology (Figures 1, 2; Supplementary Figure 1). Thus, PIC is a useful technique for spatial transcriptome analysis, with high spatial resolution and deep sensitivity without tissue loss, even in environments showing complex structures such as injured or diseased tissues.

### 4.2 Neuroblasts migrating in the peri-injured cortex have downregulated expression of genes involved in proliferation and maintenance of the immature state

Neuroblasts migrating in the RMS maintain an immature state during their long journey to the OB (Francis et al., 1999). However, our GO analysis (Figure 2) showed that migrating neuroblasts in the peri-injured cortex exhibited downregulated expression of genes involved in regulation of the Wnt and BMP signaling pathways, which are known to suppress neuronal differentiation (Wodarz and Nusse, 1998; Le, 2022). In fact, several genes associated with GO terms relevant to the negative regulation of nervous system development and differentiation were significantly decreased in cortical neuroblasts (Figure 2), suggesting that differentiation of neuroblasts is promoted in the injured cortex. In support of this finding, transplantation of NSCs derived from human-induced pluripotent stem cells in the injured brain results in rapid differentiation into neurons and glial cells (Jensen et al., 2013). Similar to our results (Figure 3), studies have reported that approximately 15–20% of neuroblasts migrating in the V-SVZ/RMS of young adult mice are Ki67-positive and are actively proliferating (Ikeda et al., 2010). In this study, we found that the expression of cell proliferation markers such as *Ccnd2*-mRNA and Ki67 protein was downregulated in cortical neuroblasts (Figures 2, 3). In general, a negative relationship exists between cell maturity and proliferative ability in neuronal lineage cells (Miyashita et al., 2021). Taken together, these findings suggest that cortical neuroblasts have a decreased ability to maintain their immature state, with a concomitant decrease in proliferative activity.

### 4.3 Neuroblasts migrating in the peri-injured cortex have upregulated expression of genes involved in control of the migration direction and prevention of cell death

In the normal brain, the migratory direction of V-SVZ neuroblasts is controlled by attractants such as prokineticin-2, netrin, GDNF, and HGF, which are secreted from the OB (Ng et al., 2005; Paratcha et al., 2006; Wang et al., 2011; Nakajima et al., 2021). However, cortical neuroblasts in the peri-injured areas are recruited by activated microglia/macrophage-derived factors in the lesion, such as osteopontin and CXCL12 (Miller et al., 2005; Yan et al., 2009). These lesion-derived attractants function through activation of integrin *β*1 signaling (Yan et al., 2009). Consistent with previous reports, our GO analysis showed that cortical neuroblasts exhibit upregulated expression of genes involved in chemotaxis and integrin signaling pathways (Figure 2). In addition, cytoskeletal reorganization is required to control the direction of neuroblast migration. The DEG analysis in this study (Figure 2) showed that cortical neuroblasts had upregulated expression of Lpar1 and Mtor, which are involved in reorganization of the actin cytoskeleton (Sarbassov et al., 2004). These results suggest that cortical neuroblasts undergo a dynamic cytoskeletal reorganization, followed by a change in migratory direction.

In the lesion, activated glial cells secrete neurocytotoxic cytokines. For example, IL-1*β* induces delayed neuronal death by ferroptosis, a non-apoptotic type of cell death caused by iron-dependent reactive oxygen species accumulation after stroke (Dixon et al., 2012; Karuppagounder et al., 2016; Zille et al., 2017). Removal of reactive oxygen species by glutathione can effectively block ferroptosis (Yang and Stockwell, 2016; Alim et al., 2019). Gclm and Slc1a1, which showed upregulated expression in cortical neuroblasts in our DEGs analysis (Figure 2), are molecules involved in the glutathione synthesis pathway and prevent ferroptosis through glutathione production (Gipp et al., 1995; Aoyama et al., 2006; Stockwell et al., 2017). Another gene with upregulated expression, Mef2c (Figure 2), also contributes to neuronal survival by inducing the expression of PGC1a, which is involved in energy metabolism in mitochondria (Ambasudhan et al., 2013). Together, these findings suggest that cortical neuroblasts have acquired the ability to migrate toward the site of injury while resisting the surrounding environment, where inflammation makes survival difficult.

### 4.4 Proliferative activity of neuroblasts migrating in the peri-injured cortex is suppressed by TGF-*β* secreted from glial cells

The GO analysis revealed that the TGF-*β* signaling pathway is enhanced in neuroblasts in the peri-injured cortex (Figure 2), suggesting that these neuroblasts are exposed to TGF-*β* in the peri-injured cortex. Previous studies have reported that TGF-*β*1 production is enhanced after stroke and traumatic injury and that its main source is activated microglia/macrophages (Lindholm et al., 1992; Lehrmann et al., 1998). Similarly, in the neonatal mouse model of cryogenic cortical injury used in this study, Iba1-positive microglia/macrophages in the injured cortex expressed TGF-*β*1 (Figure 4).

TGF-*β* binds to its receptor, the type I/II TGF-*β* receptor complex, which subsequently induces phosphorylation of the intracellular signaling molecules Smad2/3. Phospho-Smad2/3 form a complex with Smad4, which translocates into the nucleus and induces expression of genes such as p15/CDK inhibitor-2B and p21/CDK inhibitor-1, thereby inhibiting cell proliferation (Li et al., 1995; Datto et al., 1995; Hachana and Larrivée, 2022). TGF-*β* has been reported to negatively regulate neural stem/progenitor cell proliferation. For example, TGF-*β* reduces BrdU- and Ki67-positive proliferative cells in cultured neurons derived from the embryonic hippocampus and cortex (Vogel et al., 2010) and inhibits the formation of neurospheres derived from adult rat V-SVZ cells (Wachs et al., 2006). However, the effects of TGF-*β* on neuroblasts migrating in the postnatal brain were completely unknown. Our immunohistochemical studies revealed that neuroblasts express type I and type II TGF-*β* receptors (Figure 4). Furthermore, our *in vitro* experiments directly demonstrated that neuroblast proliferation is suppressed by TGF-*β* (Figure 5). These findings suggest that TGF-*β* secreted by cells in the injured cortex may be involved in the inhibition of proliferative activity of cortical neuroblasts.

In conclusion, we found that migrating neuroblasts exhibit slightly but distinctly different properties depending on the environment of their journey. The information provided by this study on gene expression profiles, comparing neuroblasts migrating in the V-SVZ and in the peri-injured cortex, could contribute to further understanding of the mechanisms of neuronal regeneration and the development of central nervous system regenerative medicine.

## Data Availability

The datasets for this study can be found at the following link: https://www.ncbi.nlm.nih.gov/bioproject/PRJNA1164820.

## Footnotes

^1^ https://metascape.org/
