# Supplementary material for: High spatial resolution gene expression profiling and characterization of neuroblasts migrating in the peri-injured cortex using photo-isolation chemistry
Source: Front Neurosci. 2025 Jan 7;18:1504047. doi: 10.3389/fnins.2024.1504047 (PMC11747130; doi:10.3389/fnins.2024.1504047)
Supplement: Supplementary file 1 [file Data_Sheet_1.DOCX]

Supplementary Material

# Supplementary Figures

**
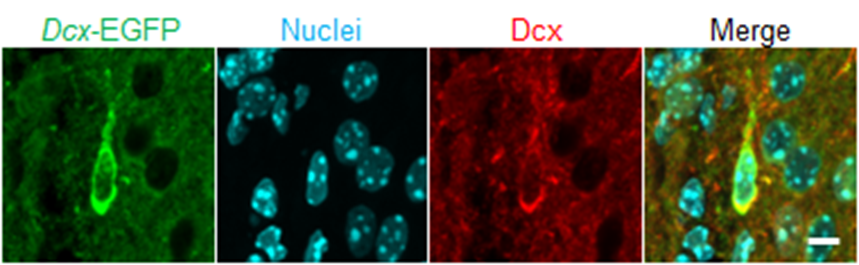
**

**Supplementary Figure 1.** Marker proteins and morphological features of UV-irradiated neuroblasts.

Immunohistochemical staining for Dcx-GFP (green) and Dcx (red) in conjunction with the Hoechst labeling of nuclei (cyan) in the peri-injured cortex. GFP-positive cells with oval nuclei were Dcx (neuroblast marker protein) positive. Scale bar, 5 μm.

**
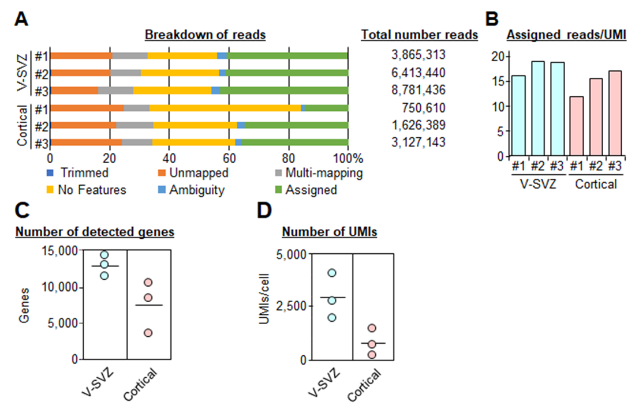
**

**Supplementary Figure 2.** Quality checks of PIC RNA sequencing.

(A) Breakdown of reads and total number of reads in the sequence results. (B) The numbers of assigned genes per unique molecular identifier (UMI). (C) The number of detected genes. (D) The number of UMIs.


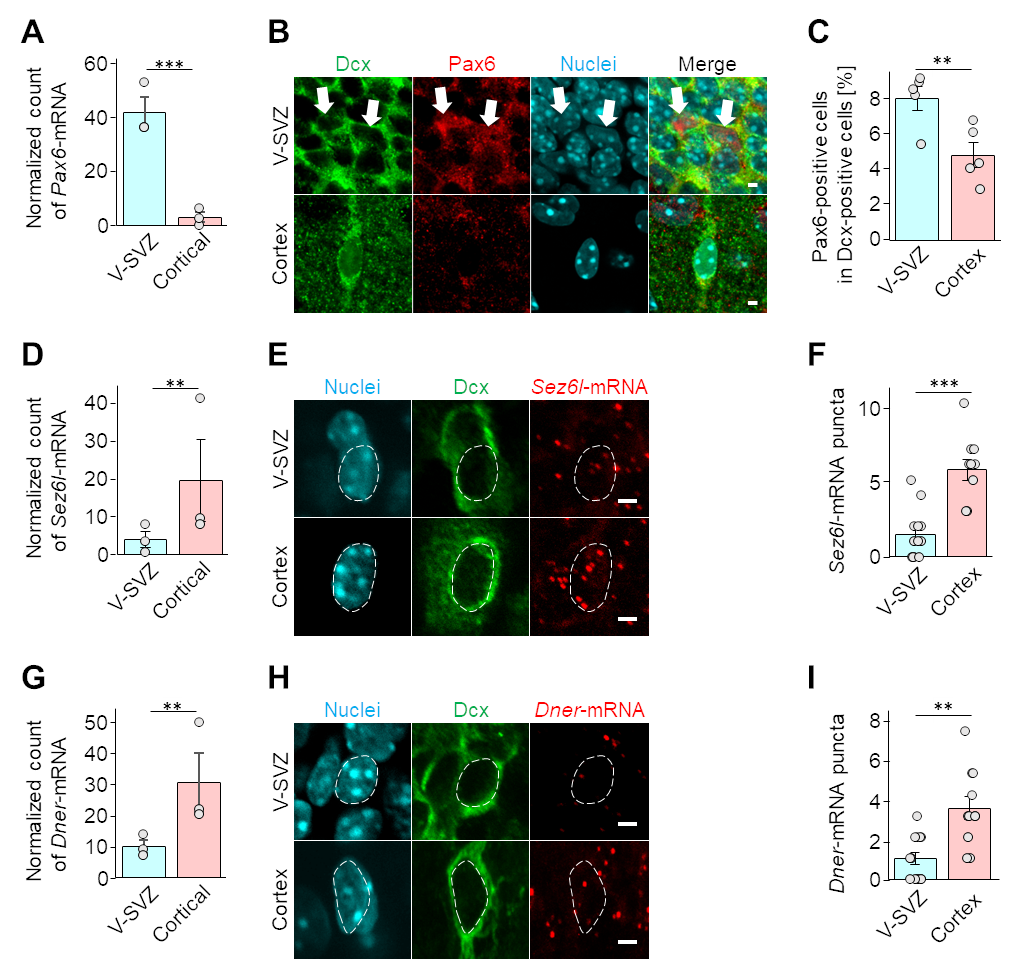


**Supplementary Figure 3.** Veracity check of the DEG dataset

(A) Normalized mRNA counts for Pax6 in PIC RNA analyses data. (B) Immunohistochemical staining for Dcx (green), Pax6 (red), and nuclei (cyan) in WT mice at 7 days post cryogenic injury. White arrows indicate Dcx-positive and Pax6-positive cells. (C) The percentages of Dcx+ cells expressing Pax6 were calculated. The graph represents the mean and SEM of 5 mice (2-3 slices per mouse). ** p<0.01, paired t-test. (D, G) Normalized mRNA counts for *Sez6l* (D) and *Dner* (G) in PIC RNA analyses data. (E, H) Fluorescence images showing in situ hybridization signals of *Sez6l*-mRNA (E, red), *Dner*-mRNA (H, red), and immunochemical staining for Dcx (green) along with nuclei (Cyan, dashed line) in V-SVZ and cortical neuroblasts. (F, I) The numbers of *Sez6l*-mRNA (F) and *Dner*-mRNA (I) puncta distributed in the nuclei in Dcx-positive cells were counted. The graphs represent the mean and SEM of 12 cells in the V-SVZ and 9 cells in the cortex of 5 mice (2-3 slices per mouse) in *Sez6l*-mRNA analyses, and 14 cells in the V-SVZ and 10 cells in the cortex of 5 mice (2-3 slices per mouse) in *Dner*-mRNA analyses. ** p<0.01, *** p<0.001, Mann-Whitney test. Scale bars, 2 μm (B, E, H).
